# Formulation and Characterization of a SIS-Based Photocrosslinkable Bioink

**DOI:** 10.3390/polym11030569

**Published:** 2019-03-26

**Authors:** Julian A. Serna, Sergio Leonardo Florez, Vivian A. Talero, Juan C. Briceño, Carolina Muñoz-Camargo, Juan C. Cruz

**Affiliations:** 1 Department of Biomedical Engineering, School of Engineering, Universidad de los Andes, Carrera 1 No. 18A-12, 111711 Bogotá, Colombia; ja.serna10@uniandes.edu.co (J.A.S.); va.talero24@uniandes.edu.co (V.A.T.); jbriceno@uniandes.edu.co (J.C.B.); 2 Department of Electrical and Electronics Engineering, School of Engineering, Universidad de los Andes, Carrera 1 No. 18A-12, 111711 Bogotá, Colombia; sl.florez10@uniandes.edu.co

**Keywords:** 3D bioprinting, decellularized extracellular matrix, bioink formulation, riboflavin

## Abstract

Decellularized extracellular matrices (dECMs) represent a promising alternative as a source of materials to develop scaffolds that closely mimic the native environment of cells. As a result, dECMs have attracted significant attention for their applications in regenerative medicine and tissue engineering. One such application is 3D bioprinting, in which dECMs can be used to prepare bioinks with the biomimicry attributes required for regeneration purposes. Formulating bioinks is, however, challenging, due to difficulties in assuring that the printed materials match the mechanical properties of the tissue which is to be regenerated. To tackle this issue, a number of strategies have been devised, including crosslinking methods, the addition of synthetic materials as excipients, and the use of synthetic matrices for casting. We are particularly interested in extrusion-based 3D bioprinting, mainly due to the ease of rapidly conducting tests for adjusting operating conditions such that the required rheological and mechanical properties are met when using it. Here, we propose a novel bioink that consists of an acid-based precipitation of a small intestinal submucosa (SIS) dECM. The formulated bioink also relies on photocrosslinking reactions to attempt to control gelation and ultimately the mechanical properties of the extruded material. Photoinitiation was explored with the aid of varying concentrations of riboflavin (RF). Manual extrusion and rheological flow tests confirmed the printability and shear-thinning behavior of all formulations. Photocrosslinking reactions, however, failed to promote a substantial increase in gelation, which was attributed to considerable entanglement of undigested collagen molecules. As a result, pendant amine groups thought to be involved in the photo-mediated reactions remain largely inaccessible. In silico computational fluid dynamics (CFD) simulations were implemented to determine shear stress values on the bioink along the exit of the printing nozzle. Moreover, we calculated a stability parameter as a means to estimate changes in the bioink stability during the extrusion process. Future studies should be directed toward assessing the role of temperature-induced gelation in the rheological properties of the bioink and the development of strategies to improve the efficiency of photocrosslinking processes.

## 1. Introduction

3D bioprinting has recently arisen as a promising biofabrication technique for the development of functional tissues and organ-like constructs in vitro. Among the different 3D bioprinting methods available, extrusion is perhaps the most used and studied. This is mainly due to the ease of printing materials within a wide range of viscosities while maintaining high cell densities when using it [1,2]. Moreover, a number of gelation methods can be supported by this technology [3]. 

One of the major drawbacks of extrusion-based 3D bioprinting is the lack of highly bioactive and mechanically suitable bioinks, which are crucial attributes used to mimic the native environment of cells [4,5]. Natural materials such as collagen, alginate, gelatin, and cellulose are preferred over synthetic ones for bioink formulation because of their high biocompatibility. One important challenge when incorporating natural polymers into bioinks is their lack of mechanical and structural stability, which can be translated into both poor printability [5,6,7] and deficient shape fidelity of the resulting constructs [8]. Printability is a measure of the ease of extruding a bioink through a nozzle or a needle without causing detrimental changes to its structure [5,6,7]. Shape fidelity is related to the ability of the printed construct to be maintained over time, a desired 3D topology under conditions of interest [8]. An avenue through which to overcome these issues is to mix them with synthetic polymers such as polyethylene glycol (PEG), polylactic acid (PLA), and polycaprolactone (PCL), which have demonstrated their ability to alter the mechanical response upon blending. Additionally, they have been proven able to shorten degradation rates, though at the expense of decreasing their biocompatibility [9].

A subclass within the natural materials group that has attracted considerable attention over the past two decades is the category of decellularized extracellular matrices (dECMs). This is due to the ability of dECMs to closely mimic the native environment of cells in vivo [10]. These materials feature a variety of structural proteins, including collagen, elastin and laminin, glycosaminoglycans, proteoglycans, and growth factors [11]. As in the case of other natural materials, one of the main limitations of dECMs is still their loose structural stability. This is disadvantageous for 3D bioprinting and is complicated even further by the complex and lengthy fabrication protocols involved in their preparation [10]. Despite these obstacles, several research groups worldwide have attempted the development of bioinks based on dECMs [12,13,14,15]. For inducing gelation, these studies have incorporated thermally-induced or photocrosslinking mechanisms, as well as a combination of the two. Despite the crosslinking strategy implemented, the achieved mechanical stability has been observed to still not be sufficient, thereby requiring the use of synthetic materials as structural improvement supports [13,14].

In photocrosslinking schemes, irradiation with ultraviolet (UV) light is the most widely used method for increasing the level of stiffness of bioinks [16]. This approach is advantageous mainly because it is minimally invasive and provides the possibility of tailoring the crosslinking density and matrix stiffness of a construct. This can be achieved by simply controlling the spatiotemporal application and intensity of light within a functional wavelength [3,16]. In spite of these benefits, UV irradiation of the bioprinted construct, particularly for the range 290–400 nm, is highly likely to detrimentally impact cell viability. This is actually the case with UV-B light (290–320 nm), which can activate the death receptor CD95 and ultimately induce apoptosis [17]. Additionally, irradiation with UV-A light (320–400 nm) may cause gene mutations [18]. Finally, the penetration depth is known to be rather short, which can ultimately lead to a significant reduction in the overall stiffness of the material [3,16]. Photocrosslinking processes rely on photoinitiators to promote reactions in response to light stimulus. Some of the most popular photoinitiators include Irgacure 2959, riboflavin (RF), and lithium phenyl-2,4,6-trimethylbenzoylphosphinate (LAP), which exhibit absorption peaks in the range 290–400 nm [16].

In this work we propose a photocrosslinkable small intestinal submucosa (SIS) dECM-based bioink with RF as the photoinitiator. The use of RF has been previously reported for collagen- or dECM-based bioinks irradiated with UV light [12,14,19]. Contrary to previously reported works, where enzymatic digestion was used to promote solubilization of dECMs, here we relied on an acid-based precipitation of the material prior to formulating the bioink. Additionally, we implemented visible light for the photocrosslinking. We hypothesized that preserving the native structure of collagen by not carrying out enzymatic digestion would yield bioinks with more suitable mechanical and degradation properties for certain 3D bioprinting applications. In addition, visible light (>405 nm) might be able to reduce the loss of cell integrity and viability reported when using UV light. Moreover, the obtained bioprinted constructs are thought to exhibit more homogeneous mechanical properties due to their greater penetration depth of visible light [16].

## 2. Materials and Methods

### 2.1. SIS Obtention and Decellularization

Four porcine small intestines were obtained from a local butcher shop and washed with tap water to remove any impurities. SIS was obtained by mechanical removal of the tunica serosa, muscularis externa, and tunica mucosa layers, and decellularized following the protocol previously described by Sánchez-Palencia et al. [20]. Briefly, the tissue was chemically treated with a proprietary solution consisting of sodium hypochlorite and hydrogen peroxide (Sigma-Aldrich, St. Louis, MO, USA) and washed thoroughly with PBS 1X and autoclaved type II water under agitation. SIS powder was obtained after drying at room temperature in a laminar flow hood and subsequently pulverizing with the aid of a freeze-miller (6875 Freezer/Mill^®^, SPEX SamplePrep, Metuchen, NJ, USA).

### 2.2. Bioink Formulation

SIS powder was solubilized at a 1.5% (*w*/*v*) concentration in 0.5 M glacial acetic acid (J. T. Baker Chemical Company, Phillipsburg, NJ, USA) for 48 h under constant agitation at 37 °C. The resulting pre-gel was then neutralized with dropwise addition of a 5 M sodium hydroxide solution (Sigma-Aldrich, St. Louis, MO, USA), lyophilized (FreezeZone Triad Cascade Benchtop Freeze Dryer, Labconco ™, Kansas City, MO, USA), and pulverized by freeze-milling. The obtained powder was solubilized in PBS 1X at a 0.32 mg/mL concentration and stirred in a magnetic plate at RT to prepare the bioink. RF powder (Sigma-Aldrich, St. Louis, MO, USA) was added to the mixture as a photoinitiator at concentrations ranging from 0.1% to 1% (*w*/*v*). The obtained mixtures were protected from light and heat. For all experiments, samples were prepared under room temperature conditions, stored in 5 mL syringes, and kept on ice until further use.

### 2.3. Sample Irradiation

A 5 W blue light LED emitting between 420 and 460 nm was purchased from a local electronics shop. As a power supply, four 1.5 V AA alkaline batteries were connected in series with a battery holder. The LED was connected to the power supply through a push button on/off switch.

The irradiance intensity of the device was explored at varying distances between the source and the sensor using a Compact Power and Energy Meter Console PM100D with a Standard Photodiode Power Sensor S121C (Thorlabs Inc., Newton, NJ, USA) (Supplementary Figure S1). For all experiments involving photocrosslinking, samples were exposed to an irradiation dosage of 3.47 J/cm^2^.

### 2.4. Rheological Characterization

Flow and time sweep experiments were performed to determine the rheological behavior of the bioinks and to assess changes in the storage (G’) and loss (G’’) moduli, before and after exposure to blue light. All experiments were performed with a parallel plate geometry (20 mm) and at 22 °C (Discovery Series Hybrid Rheometer-1, TA Instruments, New Castle, DE, USA). Flow sweep was performed between 0.01 and 200 Hz without exposure to blue light. Time sweep was performed at 1 Hz and 1% strain by maintaining a 1 mm gap. For each formulation, G’ and G’’ were measured for 180 s. The same experiment was repeated for the samples in the presence of irradiation.

### 2.5. Thermal Stability Analyses

Differential scanning calorimetry (DSC) and thermogravimetric analysis (TGA) (Simultaneous TGA/DSC SDT Q600, TA Instruments, New Castle, DE, USA) were performed to assess differences in the crosslinking degree of the different formulations after irradiation. Experiments were conducted between room temperature and 250 °C with a 10 °C/min ramp under a nitrogen atmosphere.

### 2.6. Computational Fluid Dynamics (CFD) Simulations

The velocity and shear stress distribution profiles during extrusion of the developed bioinks were determined via CFD simulations implemented in the software COMSOL Multiphysics^®^. The bioink was modeled as a non-Newtonian fluid with the aid of a power law model with parameters *n* = 0.187 and *K* = 55.160 recovered from the rheology experiments. A 2D axisymmetric simulation domain was designed according to the geometries of commercial printing nozzles 23 G, 25 G, and 27 G of the bioprinter manufacturer company Cellink^®^ (Gothenburg, Sweden). The PARDISO solver and 36,000 tetrahedral elements were required to solve the equations and assure mesh convergence. The study was conducted under steady-state conditions. A parametric analysis was performed which involved varying the nozzle diameter (200, 250, and 410 μm) and the extrusion pressure (from 5 to 60 kPa in steps of 5 kPa). Shear stress and extrusion velocity profiles at the outlet were obtained as a function of the varied parameters.

### 2.7. Calculation of the Normalized Structural Parameter λ

To assess the stability of the bioinks, the models presented by M. L. Ottone et al. [21] were implemented. According to this approach, some pseudoplastic fluids have the property of decreasing their viscosity in time in response to a constant shear stress. This change in viscosity is attributed to the detrimental changes in the stability of the microstructures that make up the fluid, a phenomenon that is known as “broken-down” [22]. These microstructure changes can be quantified by the normalized structural parameter λ, where a completely built structure is λ = 1 and a completely broken-down structure is λ = 0 [22]. This λ parameter is related to the viscosity change of the fluid over time (η), as shown in Equations (1) and (2), which may be written as

(1) $$\eta =\frac{\eta_{\infty }}{{\left(1-K\lambda \right)}^{2}}$$

(2) $$K=\left(1-\sqrt{\frac{\eta_{\infty }}{\eta_{0}}}\right)$$

where η_0_ and η_∞_ correspond to the viscosity limit values at very low and very high shear rates, respectively. As a result, Equation (1) can be rewritten to obtain the following relationship (Equation (3)):

(3) $$\lambda =\frac{\left(1-\sqrt{\frac{\eta_{\infty }}{\eta }}\right)}{K}$$

The model was calibrated by collecting the instantaneous viscosity and shear rate from CFD simulations at both the center and the wall of the nozzle tip. The extrusion pressure was held constant at 20 kPa and a time ramp of 5 s was implemented to determine variations in λ as a response to changes in the viscosity of the fluid.

## 3. Results and Discussion

**Figure 1:**
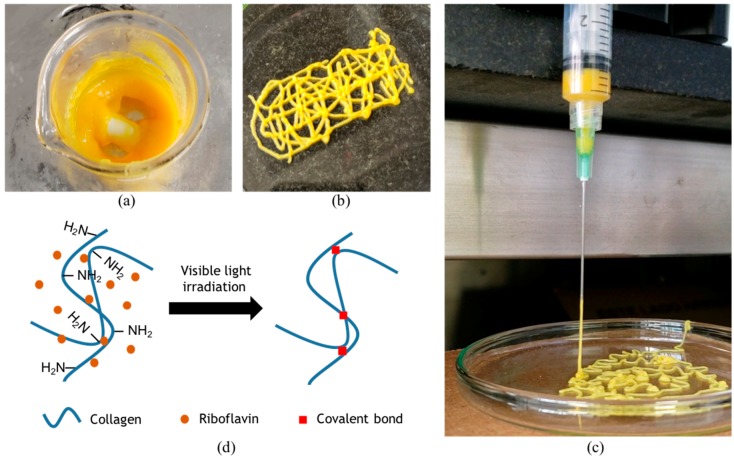
(**a**) Preparation of the 0.5% (*w*/*v*) riboflavin (RF) bioink and (**b**) its successful extrusion through a 21 G needle. (**c**) Filament formation during extrusion of the bioink through a 21 G needle. (**d**) Presumed photo-mediated crosslinking reaction thought to be occurring in the proposed bioinks.

Four different formulations of the bioink were successfully prepared. The SIS dECM concentration was held constant while RF was evaluated at four different concentrations, namely, 0%, 0.1%, 0.5%, and 1% (*w*/*v*). Extrusion tests to evaluate printability were performed with a syringe pump (Touch-Screen Syringe Pump 74905-50, Cole-Parmer, Vernon Hills, IL, USA) directly within a bioink-filled 5 mL plastic syringe equipped with a 21 G needle. The resulting mixture is shown in Figure 1a and was successfully extruded, thereby suggesting high printability levels (Figure 1b) [5]. Moreover, an actual picture of extrusion (Figure 1c) shows filament formation during extrusion, which is typically observed when bioinks exhibit sufficient printability [5]. Our experiments suggest that a successful extrusion can be accomplished while the pressure is maintained in the range 25–45 kPa.

Upon extrusion, the constructs maintained their structure in air for several days at 22 °C and 45% relative humidity. This was not the case when the constructs were submerged in an aqueous medium where structural stability was significantly reduced to about 12 h.

**Figure 2:**
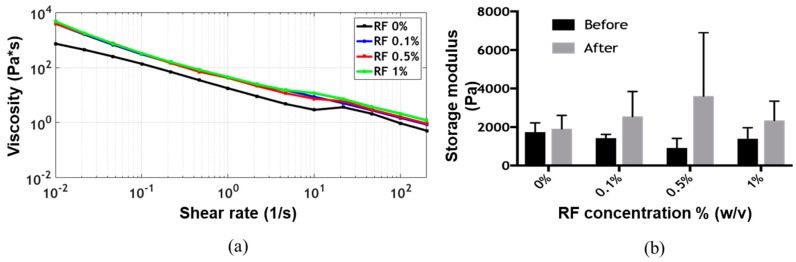
(**a**) Viscosity versus shear rate of the different formulations at 22 °C and (**b**) their storage modulus G’ before and after exposure to blue light at the same temperature.

The flow sweep showed shear-thinning behavior, which is a highly desirable characteristic of bioinks for extrusion-based bioprinting applications, for all formulations [5,9,19]. Viscosity values as a function of shear rate remained approximately at the same level for all formulations (Figure 2a). The time sweep showed gelation of the bioink before exposure to blue light, as the storage modulus G’ was always greater than the loss modulus G’’ (Supplementary Figure S2). After irradiation, the storage modulus G’ appeared to increase with the concentration of RF up to 0.5% (*w*/*v*) (Figure 2b). The presumed photocrosslinking mechanism is shown in Figure 1d. At a concentration of 1% (*w*/*v*) RF, however, the storage modulus G’ decreased, approaching the values observed for the lower RF concentrations. This was most likely due to the exceeding of the dispersion limit of RF. This phenomenon has already been reported for other RF-containing bioinks and can be attributed to mass transfer limitations resulting from photoinitiator aggregation, which ultimately lead to an increase in the photoinactive species concentration [12]. Two-way ANOVA statistical analysis for both irradiation and RF concentration showed no significant differences for the storage modulus on any of the evaluated experimental groups.

**Figure 3:**
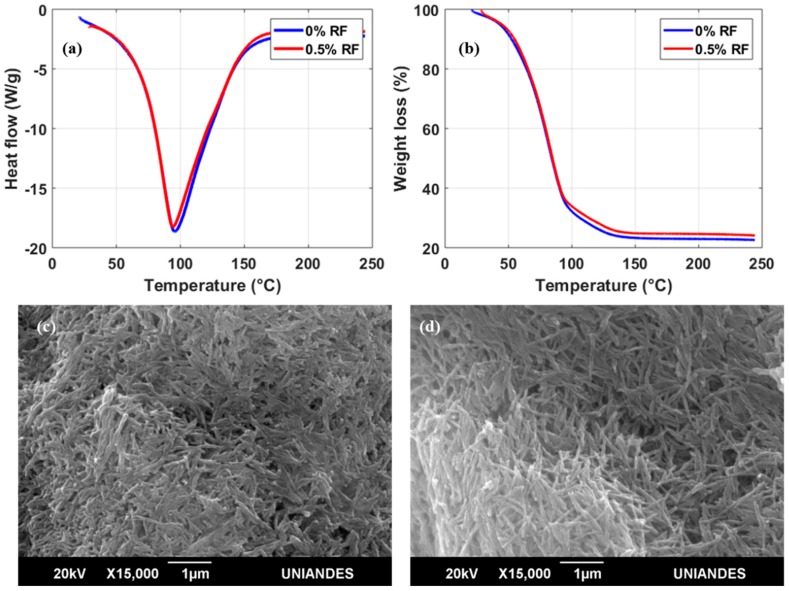
(**a**) Differential scanning calorimetry (DSC) and (**b**) thermogravimetric analysis (TGA) thermograms between RT and 250 °C after irradiation of the samples. Scanning electron microscopy (SEM) images of the bioinks at 15,000× magnification with a 20 kV accelerating voltage: (**c**) 0% (*w*/*v*) RF and (**d**) 0.5% (*w*/*v*) RF.

DSC thermograms of the bioinks in the presence or absence of RF showed only one endothermal peak; this peak is associated with the melting temperature (*T_m_*) by thermal denaturation processes of collagen (Figure 3a) [23]. For bioinks with both 0 and 0.5% (*w*/*v*) RF, the *T_m_* peak approached 96 °C. The expectation was that *T_m_* would increase in a directly proportional manner with RF concentration as a result of a higher crosslinking degree achieved after exposure to blue light [24]. These surprising results indicate that the thermal stability of irradiated collagen fibers remains unaffected by the presence of 0.5 % (*w*/*v*) RF. TGA experiments appear to confirm this finding as the weight loss profiles in the absence of RF and with 0.5% (*w*/*v*) RF are practically identical (Figure 3b). Moreover, scanning electron microscopy (SEM) images of the two bioinks (Figure 3c,d) show no apparent topographical differences at the microscale with collagen in fiber arrangements of approximately the same size. Despite the negligible changes in thermal stability in the presence of RF, a considerable increase in *T_m_* with respect to pure hydrolyzed collagen (*T_m_* = 60 °C) was still observed for our samples [25]. This was attributed to our avoidance of enzymatic treatments responsible for shortening collagen fibers during the preparation of our bioinks [25].

**Figure 4:**
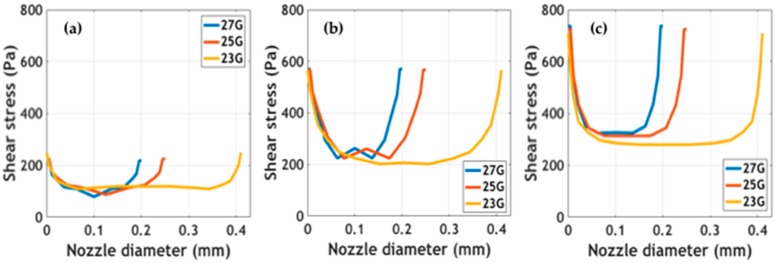
Shear stress profiles of the bioinks at different nozzle diameters and extrusion pressures: (**a**) 10 kPa, (**b**) 20 kPa, and (**c**) 60 kPa.

Simulated outlet average velocity and velocity profiles at the tip of the printing nozzles are presented in Supplementary Figure S3. Maximum velocity values were calculated at the center of the tip. Results showed that at the same extrusion pressure, maximum velocities were achieved for the larger diameter nozzle (i.e., 23 G). The impact of these velocity values on shape fidelity must be validated experimentally. Shear stress profiles at different extrusion pressures for the studied nozzle diameters are shown in Figure 4. The study of these forces being exerted on cells is of great interest to the attempt to determine the impact of the bioprinting process on cell viability [26,27]. Simulations predict maximum stress at the walls and minimum stress at the center of the nozzles. In addition, as has been reported elsewhere, shear stress appeared to increase as a consequence of incrementing extrusion pressure [27]. Blaeser and colleagues [26] have recently reported that for shear stress values below 5000 Pa, cell viability remains largely unaffected. This provides support for the notion that our bioinks can be incorporated into extrusion bioprinting processes without significant impacts on cell survival. Further experiments should be directed toward validating the collected in silico data experimentally.

**Figure 5:**
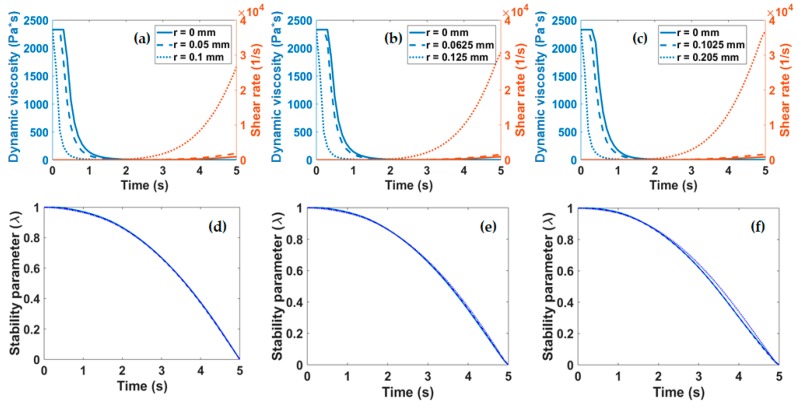
(**a**–**c**) Viscosity and shear rate as a function of time, measured at different points of the nozzle tip geometry (center, middle, and wall). Structural parameter for the three extrusion nozzles studied with diameters of (**d**) 0.21 mm, (**e**) 0.25 mm, and (**f**) 0.41 mm.

The shear-thinning behavior displayed by our bioinks as a function of time, also known as thixotropy, was studied in silico. Previous studies have already developed mathematical models to predict structural changes of biocomposites over time [21,28] and, consequently, the printability of bioinks [29]. Changes induced during the extrusion at the microscopic level which are ultimately responsible for altering rheology were assessed with the aid of the normalized stability parameter λ. This approach has been extensively used by Ottone et al. [21] to assess the stability of aqueous suspensions of collagen and was extrapolated here to our mixtures. According to the implemented model, λ = 1 corresponds to a highly stable suspension while λ = 0 describes a system where the collagen fibers assemble into spherical aggregates. The dynamic viscosity and shear rate were plotted as a function of time for the three nozzles studied in Figure 5a–c. These parameters were required to calculate λ as a function of time according to Equation (3). Figure 5d–f show that irrespective of the diameter of the nozzle employed for extrusion, λ displays a two-stage decay from 1 to 0. The first stage is characterized by a slowly changing regime that lasts for about 2 s where λ remains above 0.8. This is followed by a regime where λ changes rapidly and linearly decays to 0 over the course of about three more seconds. These findings suggest that extrusion should occur within the first stage (i.e., in about the first 2 s) to avoid collagen clustering and ultimately the clogging of the nozzle or a considerable loss in fidelity.

Supplementary Table S1 presents a comparison of reported collagen- and dECM-based bioinks against ours. Here we have listed viscosity changes for the bioinks as a function of shear rate and the highest magnitude of the storage modulus G’ achieved after crosslinking. Our bioinks feature significantly greater viscosity than those previously reported by others and have a storage modulus G’ similar to those of dECM-based bioinks for cartilage [13] and heart tissue applications [12]. Gelation for these two bioinks was, however, induced by temperature, contrary to ours where irradiation was the sole method for crosslinking. Collagen hydrogels are prone to degradation and significant structural changes for temperatures above 37 °C [11]. This supports the notion that only relatively low storage moduli G’ are attainable under such conditions, most likely due to a loss of integrity in the collagen fibers.

We hypothesize that the low photocrosslinking degree shown by the bioinks in both the rheological and thermal stability studies might be due to the entanglement of collagen fibers. Solubilization of collagen in the absence of pepsin seems to maintain the structural integrity of collagen fibers in thick staggered fibrils [30,31]. These intact native topological arrangements are thought to be responsible for the high viscosity and storage moduli observed in our bioinks prior to irradiation. This phenomenon might be also related to the presence of intact telopeptides, which are reported to facilitate intermolecular interactions between collagen fibers and to improve the stiffness and gelation kinetics of collagen-based hydrogels [32]. In our case, the entanglement of collagen most likely led to inaccessible pendant groups thought to be involved in free-radical photocrosslinking reactions.

The preliminary results shown here suggest that the developed bioink exhibits mechanical properties that are likely to assure good printability attributes. This is in line with a study by Diamantides et al. in which high values of the storage modulus G’ prior to crosslinking were correlated to exceptional printability parameters, particularly with better shape fidelity [19].

## 4. Conclusions

3D bioprinting is rapidly emerging as a powerful tool to aid the development of novel regenerative biomaterials. This is mainly due to the ability of such systems to provide a route for the preparation of customized and multifunctional constructs for regeneration of different tissues and organs. This approach needs to be enabled by a continuous supply of source materials or bioinks with inherent regenerative properties and sufficient space for tunability of key physicochemical properties. dECMs exhibit such attributes and therefore have important potential for the development of a new family of bioinks. In this work we explored the use of an SIS dECM to formulate a bioink with superior collagen integrity that is potentially able to match the mechanical properties of the most rigid tissues. This was achieved by avoiding harsh enzymatic treatments. We attempted to incorporate RF as a photoinitiator to accelerate crosslinking processes by blue light exposure but the high levels of collagen entanglement prevented its complete dispersion and ultimately a substantial impact of the photocrosslinking processes on the bioink properties. This was corroborated by DSC, TGA, and SEM analyses. Further in silico experiments allowed us to calculate a stability parameter that provided conceptual evidence for the aggregation of collagen in times as short as 5 s. Finally, rheology tests allowed us to recover power law parameters for CFD simulations that confirmed shear stress values low enough to maintain high cell viability levels. Future work will be focused on reformulating the bioink with the aid of synthetic polymers and/or thermal processing such that collagen fibers remain in an extended state and are readily accessible to the photoinitiated molecules.

## Supplementary Materials

The following are available online at https://www.mdpi.com/2073-4360/11/3/569/s1, Figure S1: Intensity versus distance from source plot for the irradiation device, Figure S2: Rheological time sweep before and after irradiation, Figure S3: Outlet velocity and velocity profiles simulated for different nozzles at varying extrusion pressures, Table S1: Comparison of similar reported bioinks in terms of rheological parameters and crosslinking strategy implemented.
