# Supplementary material for: Formulation and Characterization of a SIS-Based Photocrosslinkable Bioink
Source: Polymers (Basel). 2019 Mar 26;11(3):569. doi: 10.3390/polym11030569 (PMC6473614; doi:10.3390/polym11030569)
Supplement: Supplementary file 1 [file polymers-11-00569-s001.pdf]

Supplementary Materials

Formulation and Characterization of a SIS-Based Photocrosslinkable Bioink

Julian A. Serna [1], Sergio Leonardo Florez [2], Vivian A. Talero [1], Juan C. Briceño [1], Carolina Muñoz-Camargo [1,*] and Juan C. Cruz[1,*]

[1] Department of Biomedical Engineering, School of Engineering, Universidad de los Andes, Carrera 1 No. 18A-12, 111711 Bogotá, Colombia; ja.serna10@uniandes.edu.co (J.A.S.); va.talero24@uniandes.edu.co (V.A.T.); jbriceno@uniandes.edu.co (J.C.B.)

[2] Department of Electrical and Electronics Engineering, School of Engineering, Universidad de los Andes, Carrera 1 No. 18A-12, 111711 Bogotá, Colombia; sl.florez10@uniandes.edu.co

* Correspondence: c.munoz2016@uniandes.edu.co (C.M.-C.); jc.cruz@uniandes.edu.co (J.C.C.); Tel: +(571)-339-49-49 (ext. 1789) (J.C.C.)

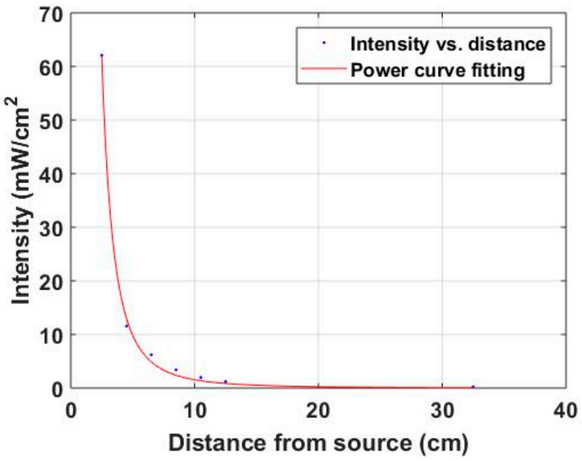

Figure S1. Dependency of the blue light irradiance intensity with respect to the distance from the sample. Data points were fitted to a power function with an R^2 of 0.98.

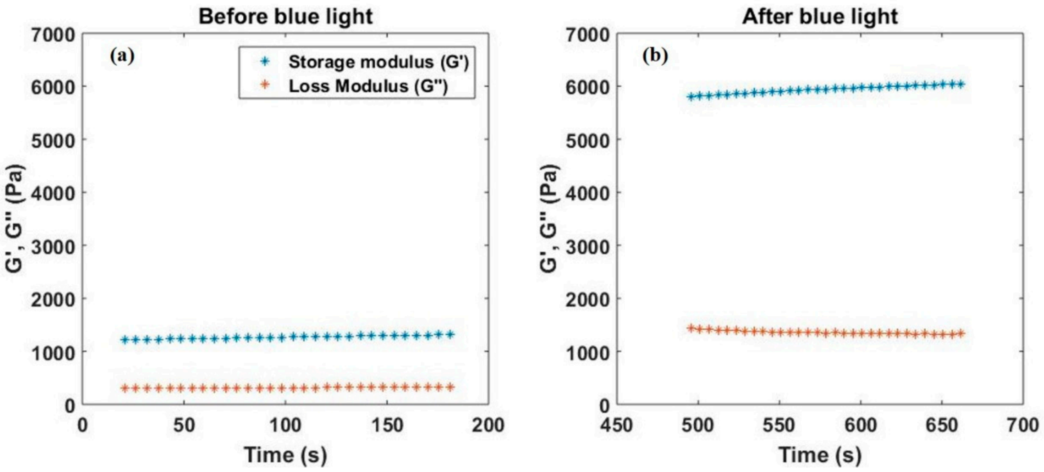

Figure S2. Rheological time sweep test for the 0.5 % (w/v) concentration of RF bioink. (**a**) Storage and loss moduli 180 s before irradiation and (**b**) after irradiation. Experiments were performed at 1 Hz and 1% strain, while temperature was held constant at 22 °C. Results showed gelation of the bioinks prior to irradiation, as the storage modulus is always greater than the loss modulus.

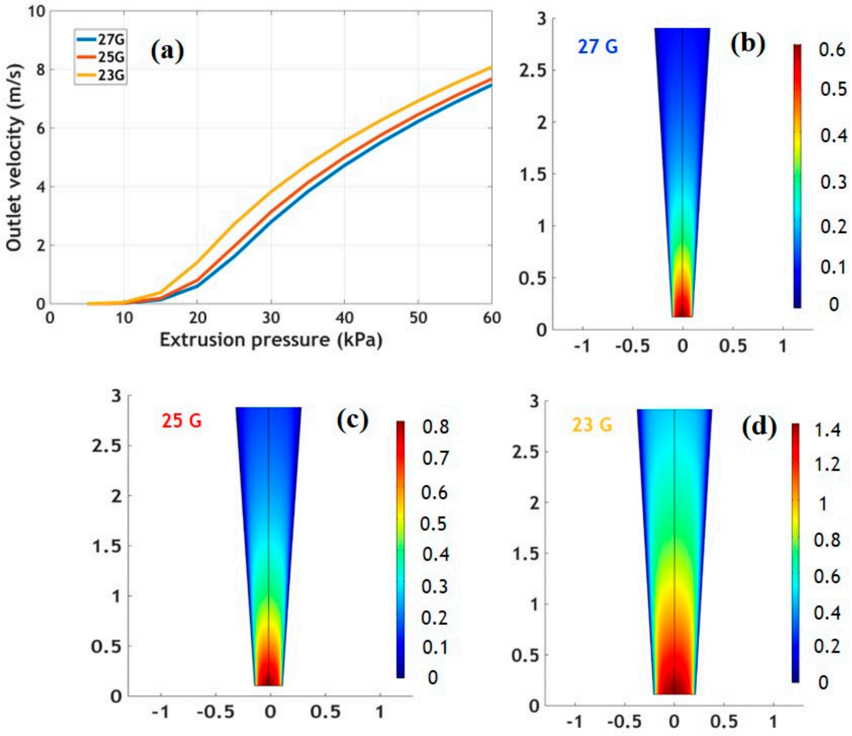

Figure S3. (**a**) Outlet velocity at the center of tip of the printing nozzle at varying extrusion pressures for three different nozzle diameters. In addition, velocity (m/s) profiles are shown for each nozzle diameter when applying a 20 kPa gradient extrusion pressure: (**b**) 27 G, (**c**) 25 G and (**d**) 23 G.

Table S1. Comparison of similar reported bioinks in terms of rheological parameters and crosslinking strategy implemented. Values displayed for viscosity correspond to flow sweep experiments between 0.01 and 200 1/s shear rate. Values displayed for storage modulus G′ are the maximum achieved after cross-linking or gelation. Disclaimer: values displayed on the table are approximations based on the figures reported by the cited authors.

Material	Gelation or crosslinking method	Viscosity (Pa*s)	Storage modulus G' (Pa)	Reference
**SIS dECM**	Photo (RF-Vis)	1.5-3900	6000	Ours
**Heart tissue dECM**	Photo (RF-UVA) and thermal	0.4-210	10,000	[1]
**Heart tissue dECM**	Thermal	0.65-35	600	[2]
**Adipose tissue dECM**	Thermal	N.A.	550	[2]
**Cartilage tissue dECM**	Thermal	0.65-2.5	4000	[2]
**Type I collagen**	Photo (RF-UVA)	N.A.	40	[3]
**Gel-MA/Type I collagen**	Photo (Ru/SPS-Vis)	N.A.	N.A.	[4]

References

1. Jang, J.; Kim, T.G.; Kim, B.S.; Kim, S.W.; Kwon, S.M.; Cho, D.W. Tailoring mechanical properties of decellularized extracellular matrix bioink by vitamin B2-induced photo-crosslinking. *Acta Biomater.* **2016**, *33*, 88–95, doi:10.1016/j.actbio.2016.01.013.
2. Pati, F.; Jang, J.; Ha, D.; Kim, S.W.; Rhie, J.; Shim, J.; Kim, D.; Cho, D. Printing three-dimensional tissue analogues with decellularized extracellular matrix bioink. *Nat. Commun.* **2014**, *5*, 1–11, doi:10.1038/ncomms4935.
3. Diamantides, N.; Wang, L.; Pruiksma, T.; Siemiatkoski, J.; Dugopolski, C.; Shortkroff, S.; Kennedy, S.; Bonassar, L.J. Correlating rheological properties and printability of collagen bioinks: The effects of riboflavin photocrosslinking and pH. *Biofabrication* **2017**, *9*, 34102, doi:10.1088/1758-5090/aa780f.
4. Lim, K.S.; Schon, B.S.; Mekhileri, N.V.; Brown, G.C.J.; Chia, C.M.; Prabakar, S.; Hooper, G.J.; Woodfield, T.B.F. New Visible-Light Photoinitiating System for Improved Print Fidelity in Gelatin-Based Bioinks. *ACS Biomater. Sci. Eng.* **2016**, *2*, 1752–1762, doi:10.1021/acsbiomaterials.6b00149.
